# *Strongyloides stercoralis* infection alters liver enzyme levels in alcoholic patients.

**DOI:** 10.7705/biomedica.7735

**Published:** 2026-06-01

**Authors:** Joelma Nascimento de Souzaa, Weslei Almeida Costa Araújoc, Alex Bruno de Souzaa, Márcia Cristina Aquino Teixeirao, Neci Matos Soareso

**Affiliations:** 1 Faculdade de Farmácia, Universidade Federal da Bahia, Salvador, Bahia, Brasil Universidade Federal da Bahia Universidade Federal da Bahia Salvador Bahia Brasil

**Keywords:** Strongyloides stercoralis, alcoholics, biomarkers, parasite load, transaminases, Strongyloides stercoralis, alcohólicos, biomarcadores, carga de parásitos, transaminasas

## Abstract

**Introduction.:**

*Strongyloides stercoralis* infection can develop into a severe and lifethreatening condition in high-risk groups, such as alcoholics. However, few studies assess the effect of these comorbidities on patients’ health.

**Objective.:**

To evaluate hematological, biochemical and hepatic biomarkers and the production of total immunoglobulin E in alcoholic patients infected with *S. stercoralis*.

**Materials and methods.:**

This was a case-control study involving 240 alcoholic patients, 60 infected and 180 non-infected with *S. stercoralis*. Evaluation of blood biomarkers was performed using automated methods, commercial kits, or nephelometry.

**Results.:**

A high frequency of low hemoglobin content was observed among alcoholics, but there was no significant difference between the infected and non-infected groups, 65.0% (39/60) and 62.8% (113/180), respectively. The frequency of eosinophilia, 58.3 % (35/60) and 26.1% (47/180) and the total immunoglobulin E concentration, 2882 and 1400 IU/ml, were significantly higher (p < 0.05) in *S. stercoralis*-infected than in non-infected individuals. ALT and AST levels were high in both groups compared to the reference values. However, the infected group presented lower AST levels, 61.5 ± 38.4 and 84.3 ± 84.6 U/L, and a lower frequency of individuals with high ALT levels, 26.7% (16/60) and 40.5% (73/180), respectively (p < 0.05). Moreover, AST levels were higher in individuals with a parasite load above 100 larvae/g of feces compared to those with less than 10 larvae/g of feces, 90.80 ± 39.9 and 56.42 ± 31.9 U/L (p < 0.05), respectively. A similar result was found for ALT.

**Conclusions.:**

This study showed fewer alterations in liver enzyme levels in *S. stercoralis*- infected alcoholic patients compared to non-infected alcoholic patients, an effect that may depend on the parasite load.

## Resumen

**Introducción.:**

La estrongiloidiasis puede convertirse en una enfermedad grave en grupos de alto riesgo, como los alcohólicos. Sin embargo, pocos estudios evalúan el efecto de esta comorbilidad en la salud de los individuos.

**Objetivo.:**

Evaluar biomarcadores hematológicos, bioquímicos y hepáticos y la producción de inmunoglobulina E total en pacientes alcohólicos infectados con *S. stercoralis*.

**Materiales y métodos.:**

Se trata de un estudio de casos y controles con 240 pacientes alcohólicos, 60 infectados y 180 no infectados con *S. stercoralis*. Los biomarcadores sanguíneos se evaluaron mediante métodos automatizados, *kits* comerciales o nefelometría.

**Resultados.:**

Se observó una alta frecuencia de alcohólicos con anemia, sin diferencia entre los grupos infectados y los no infectados, 65,0 % (39/60) y 62,8 % (113/180), respectivamente. La frecuencia de eosinofilia, 58,3 (35/60) y 26,1 % (47/180) y la concentración total de inmunoglobulina E, 2882 y 1400 UI/ml, fueron significativamente mayores (p < 0,05) en los individuos infectados con *S. stercoralis* en comparación con los no infectados. Los niveles de ALT y AST estuvieron elevados en ambos grupos. Sin embargo, en el grupo infectado se encontraron niveles más bajos de AST, 61,5 ± 38,4 comparado con 84,3 ± 84,6 U/L en no infectados y una menor frecuencia de individuos con niveles elevados de ALT, 26,7 % (16/60) comparado con 40,5 % (73/180) en pacientes infectados. Además, los niveles de AST fueron mayores en individuos con una carga parasitaria superior a 100 larvas/g de heces, en comparación con aquellos con una menor carga parasitaria (90,80 ± 39,9 y 56,42 ± 31,9 U/L (p < 0,05), respectivamente).

**Conclusiones:**

Este estudio demuestra una menor alteración de las enzimas hepáticas en alcohólicos infectados con *S. stercoralis*, lo cual podría depender de la carga parasitaria.

*Strongyloides stercoralis* is the main etiological agent of human strongyloidiasis and infects more than 600 million people worldwide, with a heterogeneous distribution and a higher prevalence in underdeveloped tropical and subtropical regions ^1^.

A peculiarity that makes this helminth so unique is its ability to multiply within the host, as the larvae can reach the infective filariform stage while still in the intestine and restart the parasitic cycle ^2^. This phenomenon is known as autoinfection and enables the parasite to remain in the host for decades without the need for exogenous reinfection, producing minimal or non-existent symptoms ^3^. However, when there is an imbalance in the host’s immune response, the infection can progress to a severe form, such as hyperinfection and/or dissemination ^4^, mainly affecting individuals co-infected with the human T lymphotropic virus type 1 (HTLV-1) ^5^^,^^6^, those in chronic use of glucocorticoids ^7^^,^^8^, and alcoholics ^9^^-^^11^.

Heavy alcohol consumption is a significant cause of mortality, morbidity, and social issues, accounting for approximately 5% of deaths worldwide ^12^. Alcoholic patients are vulnerable to several comorbidities of multifactorial etiology, resulting from the combination of malnutrition, imbalances in both the immune response and the endocrine system, and liver dysfunction ^13^. The prevalence of *S. stercoralis* in alcoholic patients varies around 20%, which is about five times higher than in the general population ^9^^,^^10^^,^^14^^-^^16^, reaching 40% in alcoholics with liver cirrhosis ^17^^,^^18^.

Chronic alcohol consumption can modulate the cellular immune response, affecting cell function, frequency, and survival, resulting in subclinical immunosuppression ^19^, which can aggravate *S. stercoralis* infection. It also leads to metabolic alterations, such as increased levels of endogenous cortisol, which in turn suppresses T cell function. In addition, cortisol increases the fecundity and survival of *S. stercoralis*, mimicking the effect of the parasitic hormone ecdysone ^20^. Additionally, heavy alcohol consumption can have a toxic effect on the bone marrow, decreasing the production of red blood cells and causing anemia, which can be aggravated by malnutrition and malabsorption of vitamin B_12_ and folic acid ^21^. Moreover, ethanol metabolism and the production of toxic substances lead to liver damage ^22^. This injury, whether acute or chronic, eventually results in increased serum concentrations of the aminotransferase enzymes alanine aminotransferase (ALT) and aspartate transaminase (AST) ^23^. This study aimed to evaluate hematological, biochemical, and hepatic biomarkers, as well as total IgE production, in alcoholic patients infected with *S. stercoralis*.

## Materials and methods

### Samples

This is a case-control study, in which 240 alcoholic patients were selected -60 infected and 180 non-infected with *S. stercoralis*- from a sample of 345 individuals ^10^.

As inclusion criteria, patients who provided three stool samples and was all the exams included in the study were enrolled. Patients with HIV, HTLV-1, hepatitis B or C, and/or those in chronic use of corticosteroids were excluded.

The selected patients were aged from 24 to 78 years and were admitted for treatment at the *Centro de Acolhimento e Tratamento de Alcoolistas* of the *Obras Sociais Irmã Dulce*, a non-governmental organization in Salvador (Bahia), Brazil, supported by the Brazilian National Health System. Patient hospitalization lasted 20 days and aimed at alcohol detoxification. Biological samples (stool and blood) were collected during the first week of admission.

### Parasitological diagnosis

Three stool samples were evaluated for each patient, collected on alternate days, using three methods: spontaneous sedimentation, Baermann- Moraes, and agar plate culture. Parasite load was measured using the Baermann-Moraes method, which was conducted following standard protocol, modified by weighing one gram of feces on an analytical balance ^9^.

### Blood biomarkers evaluation

Hematological alterations were evaluated by complete blood count (red cell count, hemoglobin content, white blood cell count, and eosinophil count) using an automated method (Sysmex™, Japan). The levels of blood glucose, total cholesterol and its fractions (HDL and LDL), triglycerides, ALT, AST, GGT, total proteins and its fractions (albumin), and total bilirubin were measured using commercial kits (Johnson and Johnson™, USA). Prothrombin time was determined by automation (Roche™, Switzerland), and IgE dosage was performed by nephelometry (Beckman Coulter™, USA).

### Statistical analysis

Statistical analyses were performed using the Statistical Package for Social Science™ (SPSS), version 21.0 for Windows (SPSS Inc., Chicago, IL, USA) and the Graph Pad Prism 9.0™ program (Graph Pad Software Inc., San Diego, CA, USA). To assess the data normality assumption, the asymmetry coefficient (skewness) and the kurtosis coefficient (kurtosis) were estimated.

A Gaussian distribution was considered when the asymmetry coefficient presented values between -1 and +1. Quantitative variables were presented using descriptive statistics (mean and standard deviation), whereas semi- quantitative variables were presented in terms of frequency. Differences between means were assessed using the unpaired t-test, to compare two groups, and differences between frequencies were assessed by Fisher’s exact test. All tests were two-tailed, and statistical significance was considered when p was less than 0.05.

### Ethical aspects

The project was approved by the research ethics committee of the *Escola de Enfermagem da Universidade Federal da Bahia*, under research number 367464. Patients were informed about the nature of the study, and those who agreed to participate signed an informed consent form.

## Results

The mean age of alcoholic individuals infected (n = 60) and non-infected (n = 180) with *S. stercoralis* was 45.3 ± 8.4 and 45.1 ± 9.5 years, respectively. The parasite load in infected individuals ranged from undetectable in Baermann-Moraes method (positive only in agar plate culture) to 793 larvae/g of feces, with a mean and standard deviation (SD) of 36.8 ± 113.9 larvae/g of feces. In addition to *S. stercoralis*, other enteroparasites were observed, with hookworms being the second most frequent, 4.6% (11/240) (table 1). No statistically significant difference was found in the frequency of coinfection with other pathogenic enteroparasites between groups (table 1).

**Table 1:** Frequency of infection by other enteroparasites in chronic alcoholic patients infected (n = 60) and non-infected (n = 180) with *Strongyloides stercoralis*

Enteroparasite	Co-infection				Total	
	Yes		No		n(%)	
	n(%)		n(%)			
Hookworms	5/60	(8.3)	6/180	(3.3)	11/240	(4.6)
*Schistosoma mansoni*	2/60	(3.3)	5/180	(2.8)	7/240	(2.9)
*Trichuris trichiura*	1/60	(1.7)	1/180	(0.6)	2/240	(0.8)
*Ascaris lumbricoides*	0/60	(0.0)	1/180	(0.6)	1/240	(0.4)
*Giardia duodenalis*	1/60	(1.7)	2/180	(1.1)	3/240	(1.2)
*Entamoeba histolytica/dispar*	*1/60*	*(1.7)*	1/180	(0.6)	2/240	(0.8)
*Endolimax nana*	2/60	(3.3)*	24/180	(13.3)	26/240	(10.8)
*Entamoeba coli*	2/60	(3.3)	0/180	(0.0)	2/240	(0.8)

* p < 0.05

Most alcoholic individuals presented hemoglobin levels below the reference value, 62.1% (149/240) (supplementary tables A and B), with a mean of 13.1 g/dl (table 2). No difference was observed between individuals infected and noninfected with *S. stercoralis*. However, the infected group had a higher frequency of eosinophilia compared to the non-infected group, 58.3 (35/60) and 26.1% (47/180) (supplementary table A) (p < 0.05), with mean eosinophil percentages of 6.6 and 3.7%, respectively (p < 0.05) (table 2). A similar result was found for total IgE concentration, in which 96.7% (58/60) and 75.1% (136/180) (p < 0.05) (supplementary table A) of infected and non-infected individuals, respectively, presented values above the reference limit (supplementary table B), with mean levels of 2,882 IU/ml and 1,400 IU/ml, respectively (p < 0.05) (table 2). Moreover, a positive correlation was found between parasite load and IgE levels (r = 0.338, p < 0.05, Pearson correlation).

**Table 2:** Mean and standard deviation of hematological, biochemical, and hepatic biomarkers and IgE levels in alcoholic individuals infected (n = 60) and non-infected (n = 180) with *Strongyloides stercoralis*

Parameter	***Strongyloides stercoralis* infection**		Total
	Infected	Non-infected	(n = 240)
	(n = 60)	(n = 180)	(X̄ ± SD)
	(X̄ ± SD)	(X̄ ± SD)	
Hemoglobin level (g/dl)	13.0 ± 1.3	13.1 ± 1.3	13.1 ± 1.3
Leukocytes (x 10^3^/mm^3^)	7.2 ± 2.4	7.7 ± 11.7	7.6 ± 10.2
Eosinophils (%)	6.6* ± 4.7	3.7* ± 4.0	4.5 ± 4.4
Total IgE (x 10^3^ UI/ml)	2.9* ± 3.3	1.4* ± 2.1	1.7 ± 2.5
Blood glucose (mg/dl)	86.0 ± 21.2	85.8 ± 21.5	85.9 ± 21.3
Total cholesterol (mg/dl)	192.8 ± 40.1	189.0 ± 49.0	190.0 ± 46.8
HDL (mg/dl)	68.7 ± 23.4	66.6 ± 27.8	67.1 ± 26.7
LDL (mg/dl)	106.1 ± 32.6	99.2 ± 34.1	100.9 ± 33.8
Triglycerides (mg/dl)	109.3 ± 114.3	114.8 ± 118.7	113.4 ± 117.4
Prothrombin activity (%)	95.9 ± 15.9	96.2 ± 17.5	96.1 ± 17.1
ALT (U/L)	38.6 ± 39.8	51.5 ± 53.2	48.3 ± 50.4
AST (U/L)	61.5 ± 38.4*	84.3 ± 84.6*	78.7 ± 76.3
GGT (U/L)	245.7 ± 317.8	322.6 ± 390.5	303.4 ± 374.4
Total proteins (g/dl)	7.26 ± 0.71	7.11 ± 0.87	7.15 ± 0.83
Albumin (g/dl)	3.98 ± 0.50	4.01 ± 0.58	4.00 ± 0.56
Total bilirubin (mg/dl)	0.85 ± 0.60	0.89 ± 0.61	0.88 ± 0.61

X̄: mean; SD: standard deviation

* p < 0.05 (unpaired t test)

Regarding blood glucose and lipid biomarkers, most alcoholic individuals had normal values, with no differences between infected and non-infected individuals. However, an atherogenic profile -that is, low HDL levels, high LDL levels, and high triglyceride concentration- was observed more frequently in non-infected alcoholics than in infected ones: 8.9% (16/180), 5.0% (9/180), and 10.6% (19/180) compared to 1.1% (2/60), 1.1% (2/60), and 6.7% (4/60), respectively, although the differences were not statistically significant (supplementary table A).

Liver biomarkers showed an altered profile. ALT and AST levels were above the reference values in both groups (table 2; supplementary table B). A significant difference was observed in the frequency of individuals with elevated levels of ALT, 26.7% (16/60) and 40.5% (73/180) (p < 0.05) (supplementary table A), and in mean AST levels, 61.5 ± 38.4 and 84.3±84.6 U/L (p < 0.05) (table 2), between alcoholic patients infected and non-infected with *S. stercoralis*, respectively. In the infected group, AST levels were higher in individuals with a parasite load greater than 100 larvae/g of feces compared to those with less than 10 larvae/g of feces, 90.80 ± 39.9 and 56.42 ± 31.9 U/L, respectively (p < 0.05) (table 3). A similar result was found for ALT levels (table 3).

**Table 3:** Serum transaminase levels in alcoholic patients infected with *Strongyloides stercoralis* according to the parasite load

Parasite load	Serum transaminase levels (U/L)		
	(mean ± SD)		
	n	AST	ALT
Non-infected	180	84.3 ± 84.6	51.5 ± 53.2
< 10	37	56.42 ± 31.9*	41.79 ± 48.16
10 to 100	17	64.12 ± 47.3	27.24 ± 12.9*
> 100	6	90.80 ± 39.9*	53.2 ± 17.2*

SD: standard deviation

* p < 0.05 (unpaired t test)

## Discussion

The pathogenesis of alcoholism is complex and involves several organic changes that make alcoholic individuals more susceptible to developing various comorbidities ^20^. The high composition of iron and polyunsaturated fatty acids in erythrocytes makes them very susceptible to damage caused by ethanol; these cells are among the most affected, after the intestinal mucosa cells ^22^. In addition, a common finding among chronic alcoholic individuals is folate deficiency, which impairs the hematopoiesis process, resulting in changes in red blood cell indices ^24^^,^^25^.

In this study, most alcoholic individuals had low hemoglobin levels, 62.1% (149/240). However, no differences were observed between individuals with and without *S. stercoralis* infection. Although *S. stercoralis* high parasite loads can cause anemia ^26^^,^^27^, the data presented here did not show an exacerbation of this condition in comorbidity with alcohol use.

No statistically significant differences were found in the mean levels of total cholesterol, HDL, LDL, and triglycerides between alcoholic patients infected and non-infected *with S. stercoralis*. However, more infected alcoholic individuals presented an anti-atherogenic profile, that is, high HDL, low LDL, and low triglyceride levels, than non-infected individuals. Inês *et al*. ^28^ had previously found an anti-atherogenic profile in alcoholic individuals infected with *S. stercoralis*.

Chronic alcohol consumption is associated with a reduction in the number, development, and maturation of immune response cells; however, it increases immunoglobulin titers, such as IgE ^29^^,^^30^. In this work, high levels of total IgE were found in alcoholic patients, being higher in those infected with *S. stercoralis* (p < 0.05). In addition, a positive correlation between total IgE levels and parasite load was observed. However, in a previous study published by our group, significantly lower levels of specific IgE antibodies were found in *S. stercoralis*-infected alcoholic patients compared to nonalcoholic infected individuals, suggesting suppression of specific IgE production ^10^. Previous studies have shown that alcohol consumption impairs antigen-presenting function and its ability to induce an antigenspecific response ^19^^,^^31^, which could explain this result.

Eosinophilia is a common finding in helminth infections, but it is generally more frequent in strongyloidiasis due to the presence of parthenogenetic females in the intestinal submucosa, which facilitates stimulation of the immune response ^32^^,^^33^. The absence of this response is usually associated with poor prognosis in immunocompromised patients ^34^. In this study, despite chronic alcohol intake, the frequency of eosinophilia was higher in patients infected with *S. stercoralis* (p < 0.05), suggesting preservation of this response.

In this work, ALT, AST, and GGT levels were altered in alcoholic patients. However, AST levels and the frequency of alcoholic patients with elevated ALT were lower in those infected with *S. stercoralis* (p < 0.05). But, when the parasite load exceeded 100 larvae/g of feces, the results were similar to those observed in non-infected alcoholic patients. This finding suggests a possible modulation of the hepatic inflammatory response caused by alcohol consumption in the presence of the parasite, which may depend on parasite load. To our knowledge, this is the first report in the literature demonstrating lower concentrations of liver enzymes in alcoholic patients infected with *S. stercoralis*. Other helminths, such as Schistosoma mansoni and Trichuris trichiura, have shown immunomodulation properties in autoimmune diseases ^35^^,^^36^.

In conclusion, in this study we observed that total IgE levels and eosinophilia were higher in alcoholic individuals infected with *S. stercoralis*. Moreover, lower AST concentrations and a reduced frequency of individuals with elevated ALT levels indicate possible protection against liver damage in alcoholic individuals infected with *S. stercoralis*, which appears to depend on parasite load.

## Supplementary files

**Supplementary table:** A. Frequency of alterations in hematological, lipid, and liver biomarkers and total IgE levels in alcoholic individuals infected (n = 60) and noninfected (n = 180) with *Strongyloides stercoralis*

Parameter		Alterations	
	Infected	Non-infected	Total
	n (%)	n (%)	n (%)
Low number of red blood cells	39/60 (65.0)	113/180 (62.8)	152/240 (63.3)
Low hemoglobin content	38/60 (63.3)	111/180 (61.7)	149/240 (62.1)
Leukopenia	5/60 (8.3)	16/180 (8.9)	21/240 (8.8)
Leukocytosis	6/60 (10.0)	21/180 (11.7)	27/240 (11.3)
Eosinophilia	35/60 (58.3)*	47/180 (26.1)*	82/240 (34.2)
High levels of total IgE	58/60 (96.7)*	133/180 (73.9)*	191/240 (79.6)
High blood glucose	8/60 (13.3)	21/180 (11.7)	29/240 (12.1)
High total cholesterol	8/60 (13.3)	4/180 (13.3)	32/240 (13.3)
Low cHDL levels	2/60 (1.1)	16/180 (8.9)	18/240 (7.5)
High levels of cLDL	2/60 (1.1)	9/180 (5.0)	11/240 (4.6)
High cVLDL levels	3/60 (5.0)	24/180 (13.3)	27/240 (11.3)
Elevated triglyceride levels	4/60 (6.7)	19/180 (10.6)	23/240 (9.6)
Increased prothrombin activity	3/60 (5.0)	10/180 (5.6)	13/240 (5.4)
Elevated alkaline phosphatase levels	6/60 (10.0)	16/180 (8.9)	22/240 (9.2)
High ALT levels	16/60 (26.7)*	73/180 (40.5)*	89/240 (37.1)
High AST levels	37/60 (61.7)	122/180 (67.8)	159/240 (66.3)
High GGT levels	52/60 (86.7)	159/180 (88.3)	211/240 (87.9)
Low total protein levels	7/60 (11.7)	29/180 (16.1)	36/240 (15.0)
Low albumin levels	7/60 (11.7)	22/180 (12.2)	29/240 (12.1)
Elevated globulin levels	15/60 (25.0)	43/180 (23.9)	58/240 (24.2)
High albumin/globulin ratio	3/60 (5.0)	16/180 (8.9)	19/240 (7.9)
Elevated total bilirubin levels	9/60 (15.0)	35/180 (19.4)	44/240 (18.3)
Elevated levels of direct bilirubin	7/60 (11.7)	29/180 (16.1)	36/240 (15.0)
Elevated indirect bilirubin levels	5/60 (8.3)	30/180 (16.7)	35/240 (14.6)

p < 0.05, Fisher’s exact test

**Supplementary table:** B. Reference values for hematological, biochemical, and hepatic biomarkers and total IgE levels

Biomarkers	Reference values
Red blood cells (x 10^6^/ul)	4.3 - 5.7
Hemoglobin levels (g/dl)	13.5 - 17.5
Eosinophils (%)	1 - 4
Leukocytes (x 10^3^/mm^3^)	4.000 -10.000
Total IgE (x 10^3^ UI/m)	< 150
Blood glucose (mg/d)	70 - 100
Total cholesterol (mg/d)	< 200
HDL (mg/d)	> 35
LDL (mg/d)	< 100
VLDL (mg/d)	> 33
Triglycerides (mg/d)	< 150
Prothrombin activity (%)	70 - 130
Alkaline phosphatase (U/L)	27 - 100
ALT (U/L)	11 - 39
AST (U/L)	11 - 39
GGT (U/L)	11 - 50
Total proteins (g/d)	6.0 - 8.0
Albumin (g/d)	3.5 - 5.5
Globulin (g/d)	2.3 - 3.5
Albumin/globulin	1.0 - 2.5
Total bilirubin (mg/d)	0.2 - 1.2
Direct bilirubin (mg/d)	0.3 - 0.6
Indirect bilirubin (mg/d)	0.2 - 0.7
